# Biological Evaluation of New Thienopyridinium and Thienopyrimidinium Derivatives as Human Choline Kinase Inhibitors

**DOI:** 10.3390/pharmaceutics14040715

**Published:** 2022-03-27

**Authors:** Pilar María Luque-Navarro, Elena Mariotto, Marco Ballarotto, Gianluca Rubbini, Francisco José Aguilar-Troyano, Alberto Fasiolo, Archimede Torretta, Emilio Parisini, Antonio Macchiarulo, Alejandro Laso, Carmen Marco, Giampietro Viola, María Paz Carrasco-Jimenez, Luisa Carlota López-Cara

**Affiliations:** 1 Department of Pharmaceutical and Organic Chemistry, Faculty of Pharmacy, Campus of Cartuja, 18071 Granada, Spain; pilarluque@ugr.es (P.M.L.-N.); gianluca.rubbini@gmail.com (G.R.); franjo0910@gmail.com (F.J.A.-T.); albertofasiolo@gmail.com (A.F.); 2 Department of Pharmaceutical Sciences, University of Perugia, Via del Liceo 1, 06123 Perugia, Italy; marco.ballarotto@studenti.unipg.it (M.B.); antonio.macchiarulo@unipg.it (A.M.); 3 Laboratory of Oncohematology, Department of Woman’s and Child’s Health, University of Padova, 35128 Padova, Italy; elena.mariotto@unipd.it; 4 Center for Nano Science and Technology @PoliMi, Istituto Italiano di Tecnologia, Via Pascoli 70/3, 20133 Milano, Italy; archimede.torretta@hotmail.it (A.T.); emilio.parisini@osi.lv (E.P.); 5 Department of Biotechnology, Latvian Institute of Organic Synthesis, Aizkraukles 21, LV-1006 Riga, Latvia; 6 Department of Biochemistry and Molecular Biology I, Faculty of Sciences, 18071 Granada, Spain; alejandrolaso@ugr.es (A.L.); cmarco@ugr.es (C.M.); 7 Istituto di Ricerca Pediatrica (IRP) Fondazione Città della Speranza, Corso Stati Uniti 4, 35128 Padova, Italy

**Keywords:** antitumoral drug, choline kinase inhibition, choline uptake

## Abstract

Due to its role in lipid biosynthesis, choline kinase α1 (CKα1) is an interesting target for the development of new antitumor agents. In this work, we present a series of 41 compounds designed based on the well-known and successful strategy of introducing thienopyridine and pyrimidine as bioisosteres of other heterocycles in active antitumor compounds. Notwithstanding the fact that some of these compounds do not show significant enzymatic inhibition, others, in contrast, feature substantially improved enzymatic and antiproliferative inhibition values. This is also confirmed by docking analysis, whereby compounds with longer linkers and thienopyrimidine cationic head have been identified as the most compelling. Among the best compounds is **Ff-35**, which inhibits the growth of different tumor cells at submicromolar concentrations. Moreover, **Ff-35** is more potent in inhibiting CKα1 than other previous biscationic derivatives. Treatment of A549, Hela, and MDA-MB-231 cells with **Ff-35** results in their arrest at the G1 phase of the cell cycle. Furthermore, the compound induces cellular apoptosis in a concentration-dependent manner. Altogether, these findings indicate that **Ff-35** is a promising new chemotherapeutic agent with encouraging preclinical potential.

## 1. Introduction

Cancer cells use different strategies to survive and to grow rapidly in the body. For instance, it is well-known that they need a high level of glucose to cope with their energetic demands. However, other changes in the cellular metabolism, such as the abnormal lipid requirements of neoplastic cells, are key to early disorder recognition. Lipids are well-known building blocks of the plasmatic membrane in eukaryotic and some prokaryotic cells. However, the role of lipids in cancer cell progression not only relates to their scaffolding contribution in cell division, but also to their function as mitogenic agents and second messengers. 

Hernández-Alcoceba et al. [1,2] were the first to observe the deregulated synthesis of phosphatidylcholine in tumoral cells and to suggest choline kinase (CK) as a molecular target for pharmacological inhibition. As a member of the phosphotransferase family of enzymes, CK performs the first transformation reaction of choline for the synthesis of phospholipids along the Kennedy pathway. Indeed, CK, which is found in the cytosol, catalyzes the conversion of choline to phosphocholine (PCho) in the presence of Mg^2+^ and ATP as cofactors. Then, phosphocholine is further modified to produce phosphatidylcholine (PtdCho) by phosphocholine cytidylyltransferase (CCT) and diacylglycerol choline phosphotransferase 1 (CHPT1) [3]. The overexpression of some enzymes in cancer highlights their significance in the proliferation process, and many of them are abnormally expressed due to the action of a protooncogene. For instance, mutations in the protooncogene Ras that causes the protein to remain in a permanent GTP-bound state impact on cell growth regulation. This leads to specific activation of CK for lipid production, promoting tumorigenesis. High CK basal levels have been also correlated to growth factors and other oncogenes such as Src and mos [4]. Increased levels of PtdCho and tCho (total choline-containing compounds), known as *the cholinic phenotype*, have been associated with tumor progression and poor prognosis. Thanks to the easy tracking of choline-containing lipids by non-invasive PET and NMR technology [5], a plethora of different cancers, such as breast, prostate, colon, and lung, have been shown to feature an aberrant choline metabolism [6]. Thus, CK has become an attractive and promising broad-spectrum therapeutic target. 

Over the years, efforts to design selective and potent CK inhibitors have progressively intensified. Using choline as a template, several inhibitors have been synthesized in which the quaternary amine cationic charge, choline’s main feature, was maintained. Before crystal structures of the enzyme were made available, the design of inhibitors was mostly guided by SAR studies, which, over time, have allowed compounds to become progressively more efficient. Indeed, CK inhibitors have evolved from monocationic [7] to triscationic [8] molecules, featuring a diverse set of linkers and cationic heads. Different assembling parts, varying from phenyl, biphenyl, bibenzyl, to biphenetyl in the linker, and from para-substituted pyridinic [9] to quinolinic [10] heads, have been tested in the process. As a result, potent inhibitors were identified, including **TCD-717** [11], **MN58b** [12], **comp. 14** [10], **ICL-CCIC-0019** [13], and **JAS239** [14]. More recently, owing to new computational studies and to the attainment of previously elusive X-ray crystallography data (PDB: 3G15) [15], further optimization of the interaction of already active inhibitors with specific amino acids in the catalytic pocket has become possible. Remarkably, all these new inhibitors remained in the choline pocket, while little if any interaction in the ATP binding site could be observed. Entire libraries of compounds that were designed to interact in both pockets as competitive inhibitors of choline and ATP actually showed interactions only in the choline pocket [16].

The positively charged quaternary amine proved to be of key importance for binding to the protein. In contrast to other kinase inhibitors that bind into the ATP pocket, which is a common feature for the members of this family of regulatory enzymes, the designed salts can selectively interact with the choline site. The hydrophobic character of this pocket, which is mainly composed of the amino acids Tyr354, Phe361, Trp420, Trp423, Ile433, Phe435, Tyr437, and Tyr440, informed the synthesis of highly hydrophobic molecules such as **Ff-35** [17] and V [7], which, however, showed a difficulty in passively crossing the plasmatic membrane and solubility problems, respectively. Hence, to overcome these inconveniences, bioisosteric molecules were designed to improve the lipophilic–hydrophilic balance that is needed to successfully become a potential drug [18,19].

However, although their inhibitory potency against the isolated enzyme was found to be in the micromolar range, their biological response was generally not completely clear. Indeed, when tested on tumoral cells, these inhibitors were not able to interact efficiently with the cytosolic enzyme.

It is important to note that free choline uses four different groups of transporters to pass through the plasma membrane: the high-affinity transporter (CHTs), choline transporter-like proteins (CTLs), organic cation transporters (OCTs), and organic cation/carnitine transporters (OCTNs). All these transporters are present at elevated, albeit variable, levels in tumor cells, depending on the cancer phenotype. The first CK inhibitor, **HC-3**, also showed choline uptake inhibition (mainly through CHT and CTL) [20]. This dual activity has also been recently observed in several compounds synthesized by our group [7,21]. It is also possible that the observed antiproliferative effect may not be a direct consequence of enzyme inhibition but the result of an indirect deregulation of the metabolism of the cell. Indeed, endoplasmic and mitochondrial stress caused by deregulation of the lipid components results in cellular senescence without the formation of reactive oxygen species (ROS). Neoplastic cells try to produce phospholipids using alternative routes, such as via methylation of phosphatidylethanolamine, excision of sphingomyelin while producing ceramides or the sebaceous glands enzymes [22] such as stearoyl-CoA desaturase (SCD).

Finally, it should be mentioned that the scaffolding role of CK is considered to be as important as its catalytic function. Indeed, the CK inhibitor **TCD-717**, which is currently in clinical trials, was shown to bind to the enzyme at its dimer interface [23]. This suggests a structural role for the enzyme, exerted through its c-Src-mediated binding to EGFR [24,25,26].

**Figure 1:**
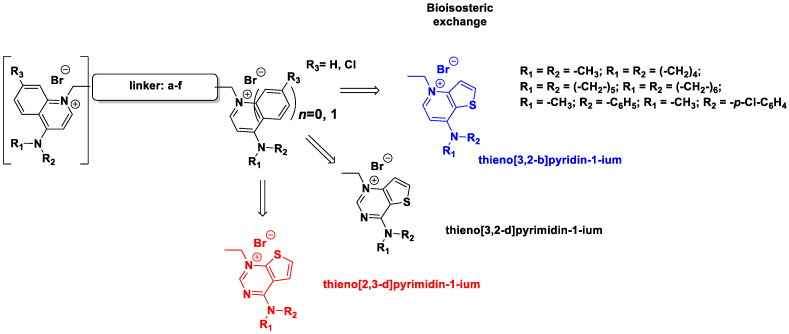
General structure of the final compounds.

In this study, we describe the biological evaluation of 41 compounds synthesized by our group as CK inhibitors. As previously described [27], these compounds were produced via bioisosteric modifications at the level of the cationic heads. While retaining previously used linker moieties, we set out to test three types of cationic heads, thieno[3,2-b]pyridine, thieno[2,3-d]pyrimidine, and thieno[3,2-d]pyrimidine (Figure 1 and Table 1) to study the effect of the bioisosteric changes on the anticancer and inhibitory activity of the compounds. The replacement of quinoline by thienopyrimidine has been extensively studied in different contexts. As a result, a large number of thienopyrimidine derivatives have been published whose antitumoral activity has been linked to the inhibition of different enzymes and to the modulation of the activity of different receptors.

## 2. Materials and Methods

### 2.1. Chemistry

The synthesis of the compounds has been described previously [27].

### 2.2. Cloning, Protein Expression, and Purification of CK

The protein was produced and purified as previously described [27]. Briefly, an N-terminally His-tagged truncated form of CKα1 (Δ75–457) was cloned into a pET-28a vector and expressed at 37 °C in *E. coli* BL21 (DE3) Star cells using 1 mM isopropyl β-D-1-thiogalactopyranoside (IPTG). After centrifugation, the cellular pellet was resuspended in 50 mM Tris-HCl pH 7.5, 500 mM NaCl, 0.2 mM phenylmethylsulphonyl fluoride (PMSF), DNase, and 0.5 mM β-mercaptoethanol, sonicated and subjected to a two-step purification procedure to isolate the target enzyme. For the first Ni-NTA affinity chromatography step, the cell lysate was first incubated for 45 min with Ni-NTA agarose bead, and then extensively washed, initially with buffer A (50 mM Tris-HCl pH 7.5, 300 mM NaCl, 10 mM imidazole) and then with buffer A + 40 mM imidazole. Protein elution was completed using buffer A + 400 mM imidazole. A second size-exclusion chromatography step was then carried out to achieve complete purity. To this end, we used a HiPrep 26/60 Sephacryl 100 HR column (GE Healthcare, Little Chalfont, Buckinghamshire, UK) and a 20 mM Tris/HCl pH 7.5, 150 mM NaCl running buffer. Overall, a total of 1.25 mg of pure protein was obtained per liter of bacterial culture.

### 2.3. Choline Kinase Assay

To study the effect of the compounds on CKα, an in vitro enzymatic assay was performed using the purified enzyme as previously reported in [19,28,29]. Briefly, the incorporation of ^14^C from [methyl-^14^C]choline into PCho in either the absence (control) or presence of different inhibitors at varying concentrations was used to determine the CK activity. The reaction mixture included 20 ng of purified CKα1, 10 mM ATP, 10 mM MgCl_2_, 100 mM Tris-HCl pH 8.5, and increasing concentrations of compounds. The mixture was preincubated at 37 °C for 5 min. Then, [methyl-^14^C]choline chloride (1 mM, 4500 dpm/nmol) was added and the reaction was left to proceed for 10 min at 37 °C. The assay was stopped by immersing the reaction tubes in boiling water. PCho was separated by Thin Layer Chromatography using methanol/0.6% NaCl/28% NH_4_OH in water (50:50:5, *v*/*v*/*v*) as solvent. The radioactivity associated into PCho was determined in a Beckman 6000-TA (Madrid, Spain) liquid scintillation counter. The 50% inhibitory concentrations (IC_50_ values) were determined from the % enzyme activity at different concentrations of synthetic inhibitors relative to the control by using a sigmoidal dose–response curve (ED50plus v1.0 software).

### 2.4. Docking Calculations

Due to the similarity of the co-crystalized ligands with the compounds under evaluation, chain A of the ligand-bound crystal structures of CKα1 (PDB codes: 4BR3 and 4CG8) were selected as structural templates for docking studies. The selected proteins were prepared for the docking step using the Protein Preparation tool included into Maestro software package (Schrödinger Release 2019-2: Maestro, Schrödinger, LLC, New York, NY, USA, 2019). The preparation process involved the addition of hydrogen atoms, the assignment of atomic bonds order as well as the protonation states of charged residues (at pH = 7). Additionally, non-structural water molecules with less than 2 hydrogen bonds with protein residues were deleted. The resulting protein structure was then minimized using the OPLS3e force field, stopping when the heavy atoms of the protein reached a Root Mean Square Distance (RMSD) of 0.3 Å. This process allowed the assignment of partial atomic charges, the refinement of the geometric parameters and the removal of steric clashes between protein residues. The ligands were prepared using LigPrep (Schrödinger Release 2019-2: LigPrep, Schrödinger, LLC, New York, NY, USA, 2019), generating protonation states at pH 7.0 ± 2.0.

The docking calculations on the prepared proteins were carried out using the Glide module (version 8.3) [29] included in the Maestro software suite. At first, each receptor structure was used to create a cubic grid centered on the center of mass of the respective co-crystalized ligands. The grid side length was set at 25 Å, while the center of mass of the ligand was set in a smaller box with a side length of 10 Å. All rotable moieties from the protein (such as hydroxyl and thiol groups of the protein residues) within the grid were set free to rotate. The selected scoring function was the SP (Standard Precision) and after sorting by the output energy score (g-score) the best 5 binding poses for each compound were selected. The best-scored binding poses for each ligand were further refined using the MMGBSA calculation from the Prime module [30], enabling movements of backbone and/or side chain of residues within 6.0 Å from the docked ligands; the ΔG_binding_ was then calculated from the obtained refined pose.

### 2.5. DFT Calculations

**Figure 2:**
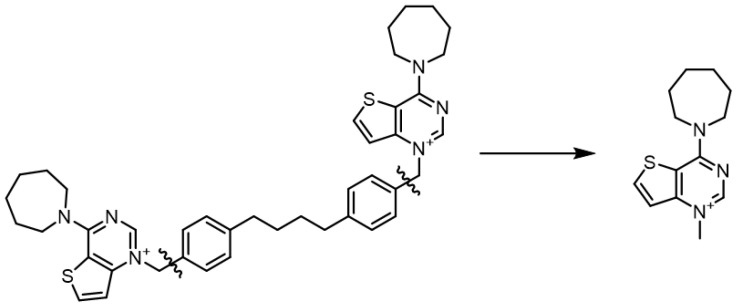
Example of the simplification of the structure to submit to the QM calculations. **Fa-29** (**left**) and the portion submitted to QM calculations (**right**).

The structures of the positively charged heads were extracted from the prepared ligands, which were previously optimized with the OPLS3e force field. Only the respective charged *N*-methylpyridinium or *N*-methylguanidinium portions were kept, discarding the linker atoms for computational simplicity (Figure 2). The resulting structures were filtered to discard duplicates and were then used as input for the Jaguar [31] module (version 10.4, release 12). The structures were minimized using DFT at the B3LYP/6-31G** level of theory and the dipole moments were calculated.

### 2.6. Antiproliferative Activity

Human cervix carcinoma (HeLa), Non-small lung adenocarcinoma (A549), Colon adenocarcinoma (HT29), and human triple negative breast cancer (MDA-MB-231) were cultured in Dulbecco’s modified Eagle’s media (DMEM). B-acute lymphoblastic leukemia (RS4;11 and SEM), T-acute lymphoblastic leukemia (Jurkat), and human promyelocytic cells (HL-60), were cultured in RPMI-1640 medium. Both media were purchased from Gibco, Life Technologies (Milan, Italy) and supplemented with 10% Fetal Bovine Serum (FBS, Invitrogen, Milan, Italy,). Every six months, each cell line was checked for the presence of mycoplasma by RT-PCR.

Stock solutions (10 mM) of the different compounds were made in DMSO. Cancer cells were seeded in tissue-treated, flat bottom, 384-well plates (Corning) according to their optimal density, (A549 1000 cells/wells; MDA-MB-231, HeLa and HT-29 2000 cells/well and finally Jurkat, HL-60 SEM and RS4;11 20.000 cells/well) in 27 µL of complete medium per well.

The day after cell seeding and immediately before cell treatment, 10 mM stock solutions of the compounds were pre-diluted to a 10 µM concentration in 250 µL of Hank’s in a 96-well plate (Corning). Subsequent 1:5 dilutions were carried out using a microlab STAR 96-CORE liquid handling system (Hamilton), a robotized liquid handling tool that was employed for all steps, from cell seeding to drug dilution and cell treatment to ensure high reproducibility while reducing time for large screening execution. This pre-dilution step was necessary to avoid DMSO-related cytotoxicity and compound precipitation in the cell culture medium. In every case, DMSO concentration never exceeded 0.1%.

Positive (Bortezomib 1 µM) and negative (0.1% DMSO) controls were included in all the screened plates to allow for monitoring the Z’ factor throughout the entire screening. Cells were treated with 3 µL of the previously made dilutions; thus, generating a 6-point 5-fold dose–response curve starting from 10 µM. Within a plate, each drug concentration was tested in duplicate in two independent experiments for each compound.

Treated cells were then placed inside the CO_2_ incubator for 72 h at 37 °C. Cell viability was assessed by resazurin assay, in which 3 µL of resazurin per well were added using the previously described automated platform. Plates were placed in the incubator at 37 °C for 2 h, and then the fluorescence of each plate was read using a Spark 10 M multimode microplate reader (Tecan Group Ltd., Mannedorf, Switzerland) with 535 nm excitation wavelength and 600 nm emission wavelength.

### 2.7. Choline Uptake Assay

Choline uptake was assessed as previously reported [7,28,29]. Briefly, HepG2 cells (200,000 cells/well) were incubated for 24 h at 37 °C with different concentrations of CKα1 inhibitors. Then, cells were exposed to [methyl-^14^C]choline (16 mM, 31 Ci/mol) for 5 min at 37 °C. The reaction was stopped by two washes with ice-cold PBS containing 580 μM choline. The cells were solubilized in NaOH 0.1 N and the total amount of radiolabel taken up by the cells was measured by liquid scintillation using a Beckman 6000-TA counter (Madrid, Spain).

### 2.8. Cell Cycle Analysis

Three different cell lines (MDA-MB-231, A549, and HeLa) were used to evaluate the effect of compound **Ff-35** on the cell cycle. Briefly, cells were treated with the test compound for 48 and 72 h. After this incubation period the cells were trypsinized, centrifuged, and fixed by adding cold ethanol (70% *v*/*v*). Cell cycles are then acquired through a cytofluorimeter (Beckman Coulter Cytomics FC500, Milano, Italy) and subsequently analyzed using MultiCycle software (Phoenix Flow Systems, San Diego, CA, USA).

### 2.9. Measurement of Apoptosis by Flow Cytometry

MDA-MB-231, A549, and HeLa cells were treated with compound **Ff-35** for 72 h and then stained using a commercial kit (Annexin-V-Fluos, Roche Diagnostics, Milano, Italy) containing Annexin-V conjugated with fluorescein isothiocyanate and Propidium iodide (PI). All the procedures were executed according to manufacturer’s instructions. Apoptotic cells were then analyzed through a cytofluorimeter (Beckman Coulter Cytomics FC500).

### 2.10. Predicted Parameters Related to the ADME (Absorption, Distribution, Metabolism, and Excretion) and PAINS (Pan Assay Interferences Structures)

The free website tool http://www.swissadme.ch/ (last accessed on 18 November 2021) allows for the in silico prediction of pharmacokinetics (gastrointestinal absorption, brain–blood barrier permeability, susceptibility by the Pgp pumping out of the cell, and cytochrome metabolism), drug-likeness (using the Lipinski rule-of-five but also Ghose, Veber, Egan, and Muegge model variations of those rules) and the medicinal chemistry friendliness (as the PAINS and structural alerts implemented by Brenk et al. [32]) of molecules under an early biological assessment. The structures of the molecules are drawn in the molecular sketcher or inserted using the SMILES format. The in silico predicted features were obtained by using free or in-house developed algorithms by the SIB Swiss Institute of Bioinformatics, which allows for a quick, robust, and easy understanding of the outcome. This helps with an early evaluation of the ADME properties of extensive libraries of compounds, which used to be the principal cause of failure at late stages of drug discovery. For that purpose, fragments were filtered in chemical libraries of compounds that are known to be unstable, reactive, toxic, or prone to interfere with biological assays, so that undesirable patterns could be recognized early in medicinal chemistry synthesis and evaluation. For further details on the algorithms and databases used, see reference [33].

## 3. Results and Discussion

### 3.1. Biological Assays

#### 3.1.1. Docking Results

**Figure 3:**
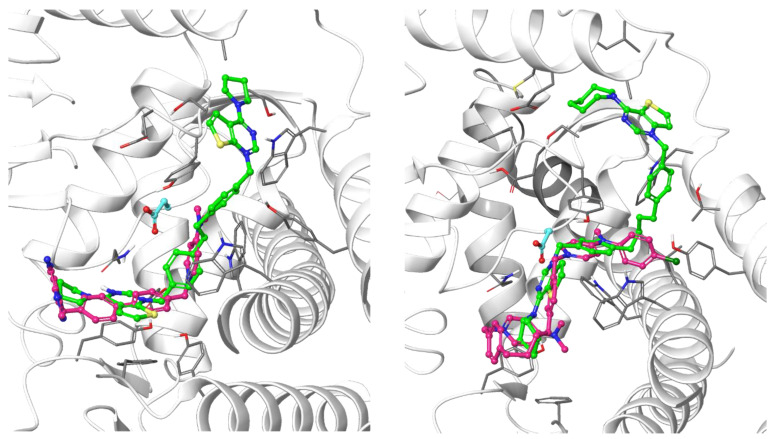
Predicted binding mode of two of the docked compounds into the crystal structures of CK, PDB codes: 4BR3 (left) and 4CG8 (right). The protein is shown as white ribbons; the respective co-crystalized ligands are depicted in magenta stick-and-balls, while the docked compounds (**Fg-15** on the left and **Fa-29** on the right) are shown as green stick-and-balls. For reference, Asp306 is shown in cyan.

All the ligands docked into the chosen protein structure are found to interact with the residues of the known choline-binding site via one of the cationic heads through π-π and cation-π interactions. In both proteins, the ligands assume an elongated conformation, different from the conformation assumed by the respective co-crystallized ligand (Figure 3). This can be rationalized by considering the difference in the structure of the linker, which depends on its length and flexibility, and the difference in size of the cationic head moiety, which varies from the small *N*,*N*-dimethylpyridinium to the much larger cycloalkyl-substituted thieno-fused nitrogen heterocycle.

**Figure 4:**
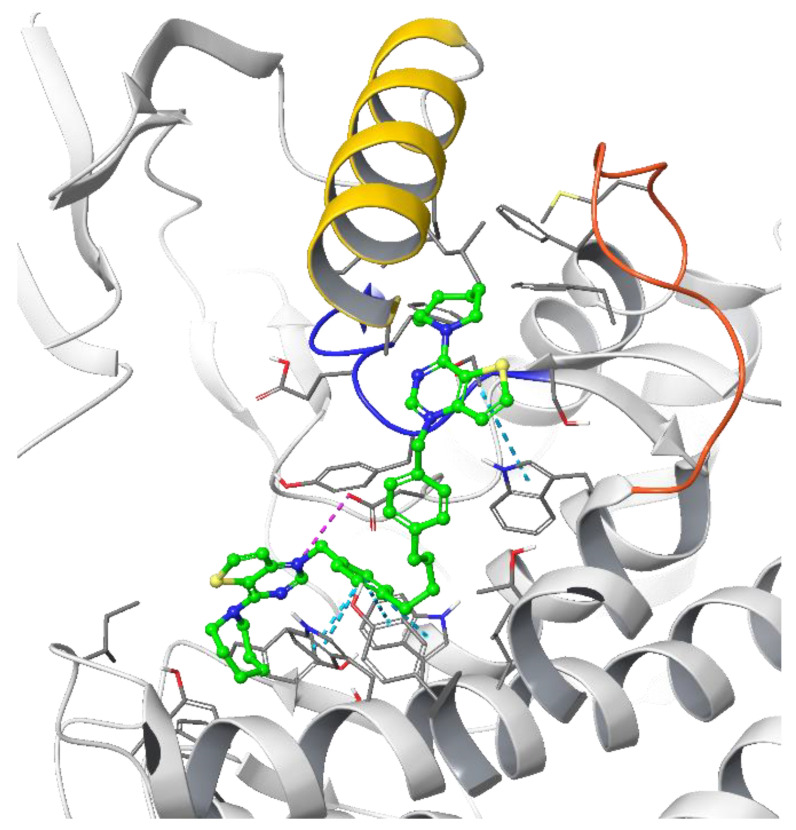
Putative binding pose of the most potent compound **Fa-29** (in green) into 4CG8 crystal structure. Ionic and π-π stacking interactions are highlighted as dashed magenta and cyan lines, respectively. The choline binding motif is represented with a blue ribbon, the B helix is in yellow and the loop connecting helices D and E is represented in orange.

The theoretical binding poses feature a number of hydrophobic interactions: in the choline-binding site, one of the charged heads and an aromatic ring of the linker strongly interact with Trp420 and Trp423, while the cycloalkyl substituent is buried into a hydrophobic cavity that in the crystal structure hosts the co-crystallized ligand. The only established polar interaction is an ionic contact of the charged head with Asp306, a key residue involved in the catalytic cycle of the enzyme. The remaining portion of the alkyl chain of the linker and the other cationic head reach a secondary binding pocket that can accommodate the bulkier cycloalkyl substituent (Figure 4). This additional pocket is formed by the hydrophobic residues from the B helix, the “choline binding motif” [34], and the loop connecting helixes D and E and while the cycloalkyl ring fits nicely into the hydrophobic pocket, the associated aromatic head is located in a slightly more polar area. The choline-binding motif contains the negatively charged residues (Glu332 and Asp330) that are involved in the binding of magnesium. A change in loop conformation due to the ligand binding could also lower the capability of the protein to bind the Mg^2+^ ion that is necessary for its biological function [15].

**Figure 5:**
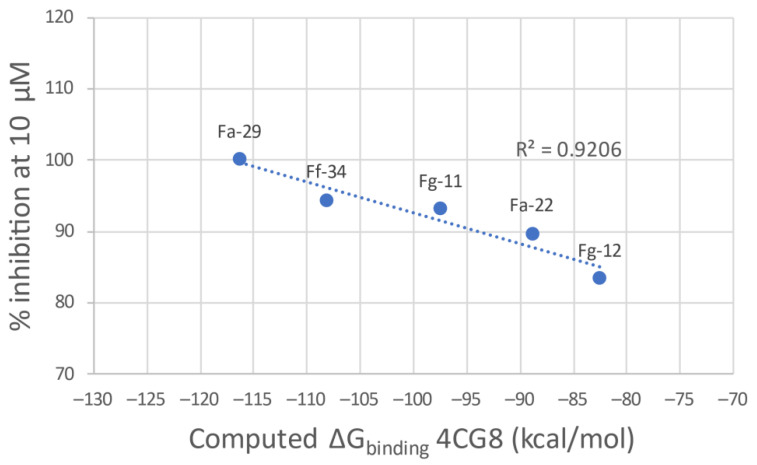
Plot of the calculated ΔG_binding_ of the most potent compound of each linker class against the experimental percentage inhibitory potency at 10 μM.

The g-scores for the two proteins are reported in Table 2. Docking into the 4BR3 crystal structure results in the best g-scores on average. However, the lack of correlation between the ligand potency and the docking score encouraged us to evaluate the ΔG_binding_ derived from a MMGBSA calculation on the best-scoring binding pose. Regrettably, also in this case the ΔG evaluation did not result in the delineation of a clear general trend. This prompted us to evaluate a more qualitative Structure–Activity Relationship (SAR) analysis. After the minimization process with Prime, 4CG8 is the protein that shows the best correlation between the computed ΔG_binding_ and the inhibitory potency of the most potent ligands for each class (Figure 5). Thus, 4CG8 was chosen for trying to build a SAR scheme from the obtained poses, comparing only the binding poses obtained from the most potent ligands for each class.

**Figure 6:**
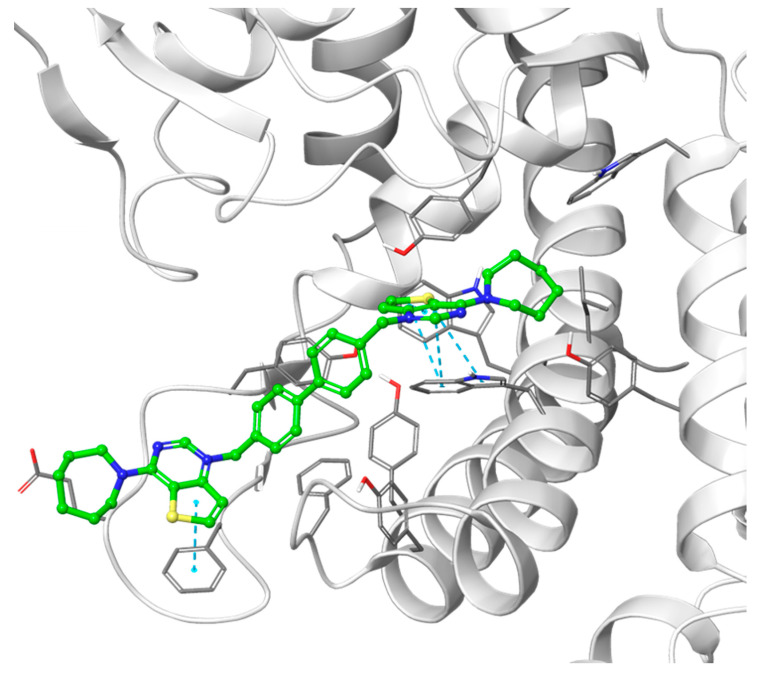
Hypothetical binding pose of the most potent compound from the biphenyl class, **Fa-22** (shown in green), into the 4CG8 crystal structure. π-π stacking interactions are highlighted as dashed cyan lines.

The predicted binding poses of the most potent compounds for each linker class (Figure 6) were then used to rationalize the Structure–Activity Relationship for the other compounds:

- Decreasing the cycloalkyl ring size (from azepane to piperidine to pyrrolidine) could reduce the occupancy of the hydrophobic pockets found in the choline binding site and near the choline binding motif.
- Changing the butyl linker to a diphenoxyethyl one could have three distinct effects on the binding pose: i. The linker could become less flexible due to the Hydrogen Bond Acceptor (HBA) nature of the added oxygen atoms, restricting the linker in a less elongated conformation; ii. The delocalization of the oxygen lone pair into the aromatic ring could increase the energy barrier for the rotation around the C_aromatic_-O bond; iii. The increased electron density in the phenyl rings could weaken the π-π interactions with the electron-rich side chains of the Trp and Tyr residues due to higher electrostatic repulsion.
- Shortening the linker from a length of 4 carbon atoms to 2 (butyl to ethyl linker) can lead to a not optimal position for the cationic head, which would not reach the secondary pocket.

Introducing shorter, more rigid linkers such as the biphenyl and the 2,2′-bipyrimidyl ones changes the predicted binding pose: while one of the charged heads is anchored into the choline binding pocket, the other cannot reach the secondary back pocket due to the low flexibility of the linker and is exposed to the solvent (Figure 6).

Although the developed SAR scheme can convincingly interpret the experimental results, some of the structural changes showed a peculiar effect on the activity. Notably, a significant reduction in the inhibitory potency was observed when a slight structural modification was introduced into the positively charged heads, which consists in the change in orientation of the fused thiophene ring from the thieno[3,2-*d*]pyrimidinium into the thieno[2,3-*d*]pyrimidinium moiety. This phenomenon was observed for every pair of compared compounds that differ only by the orientation of the thiophene, albeit to a variable extent. Thus, we were prompted to further examine the electronic properties of these aromatic portions to investigate the causes of this activity cliff (Table 3).

**Figure 7:**
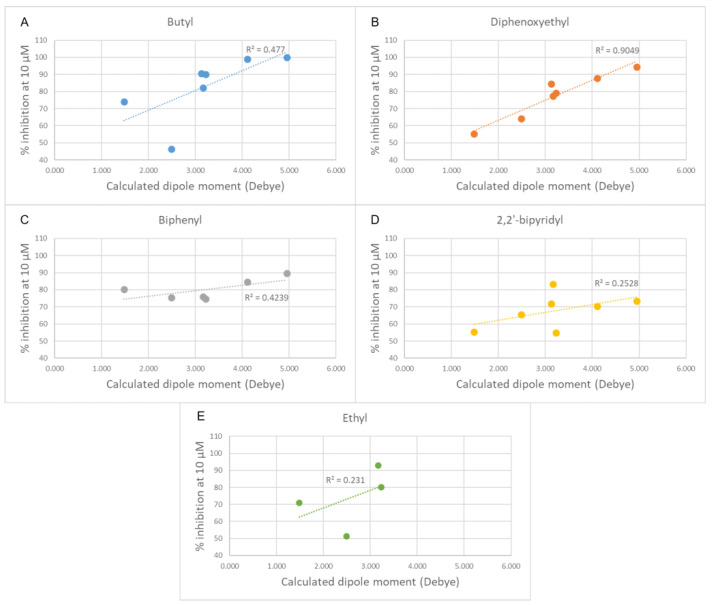
Plots of the experimental percentage inhibitory potency against the calculated dipole moments of the cationic heads for each class of compounds. (**A**) Butyl linker class. (**B**) Diphenoxyethyl linker class. (**C**) Biphenyl linker class. (**D**) 2,2′-bipyridinyl linker class. (**E**) Ethyl linker class. In each plot, the linear fit is shown as a dashed line.

**Figure 8:**
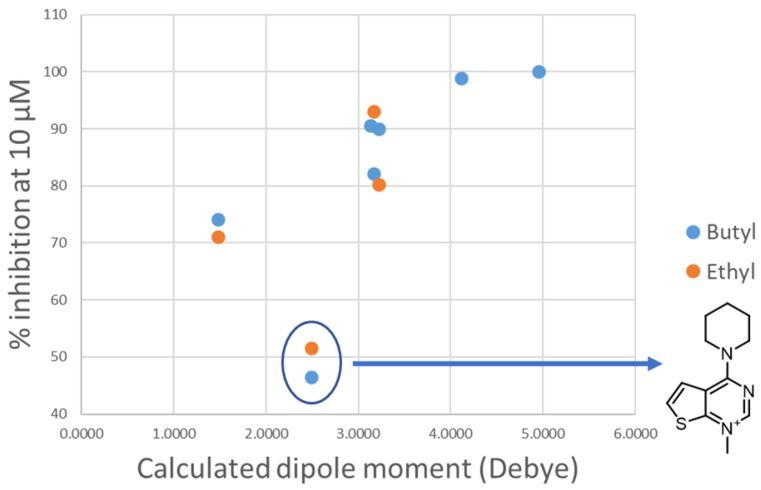
Plot of the experimental percentage inhibition at 10 μM against the calculated dipole moment of the heads for the classes of compounds containing a butyl or ethyl linker (blue dots and orange dots, respectively). The outlier head is circled in blue and the structure is reported on the right.

Changing the orientation of the thiophene ring fused to the charged heads influences their charge distribution and dipole moment. Both those features play a role in π-cation stacking [15]. The compounds were divided by linker class (butyl, ethyl, diphenoxyethyl, biphenyl, and 2,2′-bipyridyl linkers) and, for each group, the experimental inhibition potency of each compound was plotted against the calculated dipole moment of its charged head (Figure 7). Due to the low number of compounds, the high standard deviation error of their inhibition assays and the relative similarity of their potency values, the group containing the compounds featuring only a single charged head was left out of this analysis. A trend was observed in all classes of compounds examined, with various degrees of positive correlation and with inconsistencies that can be related to a different contribution of this molecular descriptor to the binding affinity. These results suggest that those heads that have a higher dipole moment can more strongly interact via π-stacking interactions with the electron-rich aromatic residues in the binding site (tryptophan, tyrosine, and phenylalanine), leading to a stronger affinity. Although for the set of compounds that contain an ethyl linker we lack the potency data for three out of the seven different charged heads, due to their scarce solubility, the available data strongly resemble the data from the butyl class (Figure 8). An outlier was identified among the compounds of the butyl and ethyl linker classes, the compound with the piperidine-substituted thieno[2,3-d]pyrimidinium head, possibly due to a conformational shift that reduces the strength of the π-stacking interactions. Removing it from the analysis considerably improved the correlation with the ligands of the butyl class (R^2^ = 0.88). The lack of a strong correlation in both the biphenyl and bipyridyl classes could be explained by a lower contribution of the head π-stacking interactions to the total binding energy, possibly due to the higher rigidity of the linker, which does not allow an optimal interaction geometry.

To determine the potency of the compounds, preliminary end-point assays were undertaken with the inhibitors at the concentrations of 10 and 30 μM. Then, the IC_50_ values of the most potent inhibitors were determined (Table 4). In general, these values are in agreement with the docking studies. For instance, the larger volume of the quaternary amine in the cationic head is still key to the activity, so the azepane ring is postulated to be the most suitable, especially when the spacer is longer. The introduction of bipyridine as a linker (Family **C**) has a negative effect on activity, with IC_50_ values higher than 25 μM. A two carbon atom increase in the distance between the two phenyl linkers in family D does not seem to have much effect on the inhibition of the enzyme in comparison to compounds with biphenyl linkers (B). However, as some of these compounds suffer from solubility problems and their IC_50_ values could not be determined, this observation cannot be fully confirmed.

Conversely, when comparing families D and E, which feature the introduction of two or four carbon atoms in the linker, a slight difference in the percentage of inhibition at 10 and 30 μM was observed, also reflected in the IC_50_ values.

However, some inconsistencies were observed when comparing the IC_50_ values with the docking data. First, the inhibitory activity decreases with shorter linkers. Thus, the IC_50_ values reveal that the biphenyl linker (B) can be one of the most effective, compounds **Fp-1** being the most active, with an IC_50_ of 1.06 μM. Again, the different solubility of the compounds may be involved in these results, particularly when considering the insolubility of compounds **Fa-23**, **Fa-25**, and **Fg-19** in this family. However, these excellent values do not translate into good inhibitory activity on cell growth.

The second inconsistency relates to the thieno[3,2-d]pyrimidinium cationic head, which, from docking data, appears to be preferable to the thieno[2,3-d]pyrimidinium one, particularly when the cycloalkylamine is piperidine, while the IC_50_ values do not support this isomer preference. Indeed, their difference appears to be the opposite when the cycloalkylamine is azepane (e.g., **Fa-29** (thieno[3,2-d]pyrimidinium) IC_50_ = 1.08 μM while **Ff-35** (thieno[2,3-d]pyrimidinium) IC_50_ = 0.46 μM and **Fa-34** IC_50_ = 0.704 μM versus **Ff-36** IC_50_ = 0.350 μM), although for the rest of the compounds a slight preference for the [3,2-d] isomers can be appreciated.

#### 3.1.2. Antiproliferative Activity and Inhibition of Choline Uptake

The results are shown in Table 4, where the compounds were grouped in six families depending on their linker moiety: monocationic compounds (A), biphenyl (B) bipyridinyl (C), bibenzyl (D), biphenetyl (E), and 1,2 diphenoxiethane (F). Each family is, in turn, subdivided based on the cationic head into thieno[3,2-b]pyridin-1-ium (blue), thieno[3,2-d]pyrimidin-1-ium (black) and thieno[2,3-d]pyrimidin-1-ium (red). Finally, each compound is substituted in position 4 or 7 by a pyrrolidine, piperazine, azepane, *N*-methylaniline, *p*-chloro-*N*-methylaniline. Table 4 summarizes the growth inhibitory effects of the compounds against eight tumor cell lines: cervix carcinoma (HeLa) cells, human colon adenocarcinoma (HT-29), B-acute lymphoblastic leukemia (RS4; 11 and SEM), human promyelocytic cells (HL-60), human T-cell leukemia (Jurkat), human non-small cell lung carcinoma (A549), and breast adenocarcinoma (MDA-MB-231). Data are shown in comparison with **MN58b** and **RSM 932A**, which were used as reference compounds.

In general, if we exclude the monocationic compounds **FMa1** and **FMa3**, the best performing compounds are those with longer linker moieties (bibenzylic (D) and biphenethyl (E) families). These data are in agreement with those previously published, where the lipophilicity of the compounds plays a crucial role in their antiproliferative activity, probably due to a facilitation effect on their passage through the cell membrane. On the other hand, it is remarkable that those families in which heteroatoms were introduced in the spacer bipyridinyl (C) and 1,2-diphenoxyethane (F) show a considerably diminished anti-proliferative activity, probably also due to their reduced lipophilicity relative to their homologues.

Starting with the family of monocationic compounds, we observed good antiproliferative activity. The compound with thieno[3,2-b]pyridin-1-ium as cationic head (**Fa-M2**) is slightly more active than the others and, coincidentally, also the most lipophilic. However, the complete lack of inhibitory activity on the enzyme suggests a different mechanism and no correlation between its antiproliferative and its inhibitory properties. Monocationic compounds can be considered as bioisosters of those recently described by our group [35], particularly the compound **s** 1-([1,10 -biphenyl]-4-ylmethyl)-7-chloro-4-(pyrrolidin-1-yl)quinolin-1-ium bromide. The monocationic compounds show an up to 10 times lower antiproliferative activity than compound **s**. This could be a consequence of the reduced lipophilicity (cLog P = 3.00–3.47) of the bioisosters with respect to compound **s** (cLog P = 3.91) but also of the reduced activity on the inhibition of choline uptake (see the last column of Table 4).

Concerning the families of biscationic compounds, the compounds with a biphenyl linker show moderate potency on cell growth inhibition. Considering the cationic head, the thienopyridine derivatives (**Fg-9** and **Fg-10**) show a slightly improved activity over the thieno[2,3-d]pyrimidinic derivatives (**Fg-14**, **Fg-30**, and **Fg-18**) while these, in turn, are significantly more active than the thieno[3,2-d]pyrimidinic derivatives (**Fa-21**, **Fa-24**, and **Fa-22**). On the other hand, it seems that the bulky cycloalkylamine has a positive influence, albeit not as noticeable as when the amine is azepane. However, the **Fp-1** and **Fp-8** compounds with *N*-methylaminiline and *p*-chloro-*N*-methylaniline provide a better antiproliferative performance than their homologues with cycloalkylamines. It is also interesting to note that family **C** with a bipyridinic spacer are the worst compounds of all those presented in this work, both in terms of enzymatic inhibition and antiproliferative properties.

Compounds belonging to the D family, which feature a bibenzylic spacer, show a remarkable increase in activity compared to the biscathionic B and C families. However, no substantial differences in activity related to the different cationic heads or to the cycloalkylamine they carry are reflected within family D, even though the thieno[2,3-d]pyrimidinic isomers seem to be slightly more active than the others. The compounds that belong to the E family, which feature a biphenethyl spacer, are those that are associated with the best antiproliferative properties. This could be due to their increased lipophilicity. Again, the bulky cycloalkylamine seems to have a positive effect, so that compounds with piperazine and azepane stand out from those with pyrrolidine, the best isomers being thieno[2,3-d]pyrimidinic. The family that features 1,2-diphenoxiethane as a linker (F) behaves similarly to the E family. However, the fact that the F family is less lipophilic due to the presence of O atoms does seem to affect its antiproliferative activity compared to other less lipophilic compounds (C family), probably due to its good enzyme inhibition values. It is worth noting that, in general, these compounds have particularly remarkable antiproliferative behavior on MDA-MB-231 and HL-60 cell lines.

Finally, among all the compounds, **Ff-35** is the most active, with a good correlation between antiproliferative and enzymatic activity.

In general, we observed a good correspondence between antiproliferative activity and enzymatic activity, although we cannot rule out that the inhibition of choline uptake may represent an alternative mechanism, as suggested by the most representative compounds of the most active families (**Fa-M1**, **Fa-22**, **Ff-35**, and **Ff-36**, see the last column of Table 4).

#### 3.1.3. Cell Cycle Analysis

Considering that **Ff-35** is one of the most active antiproliferative compounds and that this also correlates with its inhibitory activity against CK and on the choline uptake, we set out to analyze its effect on the cell cycle in three different cell lines.

**Figure 9:**
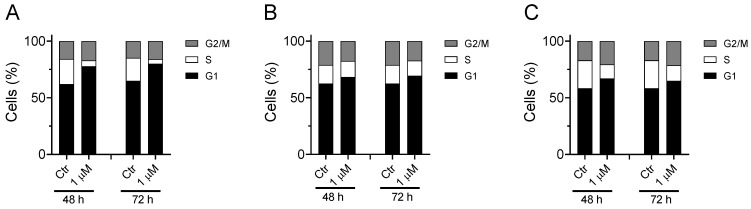
Effect of **Ff-35** on cell cycle in A549 (**A**), Hela (**B**), and MDA-MB-231 cells (**C**). Cells were treated with the compounds for 48 and 72 h, at the concentration of 1.0 µM. After this period, the cells were processed as described in the Materials and Methods section.

As shown in Figure 9, the compound induces a notable increase in the G1 phase accompanied by a reduction in the S phase. This effect can be observed in all three cell lines analyzed, although the greatest effect is observed in A549. These results are in good agreement with literature data indicating G1-phase arrest in various cell lines for choline kinase inhibitors [18,19,36,37].

#### 3.1.4. Measurement of Apoptosis by Flow Cytometry

**Figure 10:**
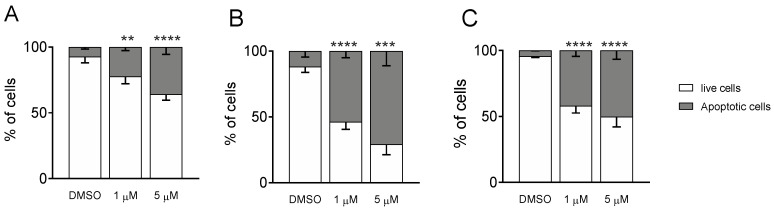
Compound **Ff-35** induces apoptosis in A549 (**A**), Hela (**B**), and MDA-MB-231 cells (**C**). Cells were treated with **Ff-35** for 72 h at the concentrations of 1 and 5 µM. The cells were then harvested and labeled with annexin-V-FITC and PI and analyzed by flow cytometry. Data are represented as mean ± SEM of three independent experiments. ** *p* < 0.01, *** *p* < 0.001; **** *p* < 0.0001 vs. DMSO.

To better decipher the effect of **Ff-35** on cell death, A549, HeLa, and MDA-MB-231 cells were labeled with both annexin-V-FITC and PI and then analyzed by flow cytometry, allowing the quantitative analysis of living cells and apoptotic cells, respectively. All three cell lines treated with **Ff-35** for 72 h at two different concentrations (Figure 10) displayed a significant increase in apoptotic cells in a concentration-dependent manner, in good agreement with the cytotoxicity data.

#### 3.1.5. Predicted Parameters Related to the ADME (Absorption, Distribution, Metabolism, and Excretion) and PAINS (Pan Assay Interferences Structures)

Herein, we report some predicted parameters related to the ADME suitability of the synthesized compounds (Table 5).

For this purpose, we used the free web tool SwissADME [32] (http://www.swissadme.ch last access the 18 November 2021) developed by the Swiss Institute of Bioinformatics.

The Log P demonstrated to correlate well with the inhibitory binding potency and was calculated as an average of five predicted methods. On the other hand, PAINS (Pan Assay Interferences Structures) [33] and the structural alert [38] give us information on molecular fragments that could lead to a potent biological response that does not correspond to the target but to an off-target cytotoxic effect. Some of those fragments are, for example, phenol-sulphonamides, enones, quinones, and catechols, and databases that can recognize such interference structures in our synthesized compounds could provide very useful information. As we can see in Table 5, the quaternary nitrogen triggers a structural alert because it makes the molecule more reactive; also, it can behave as a surfactant agent. However, this quaternary N is an essential feature to mimic the choline substrate and to bind to choline kinase.

**Figure 11:**
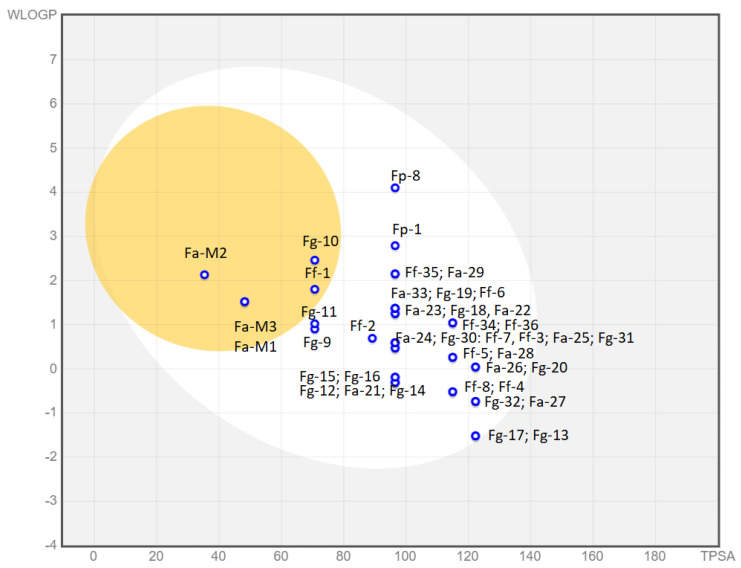
Boiled Egg chart. In the yolk, there are compounds probably permeable to the BBB. In the white part, there are those that could show GI absorption and in the outer part (in grey) those with low absorption and limited brain penetration. In Table 5, compounds are color-coded: red (not suitable), orange (partially suitable), and green (suitable).

The *Suitability* column refers to the BOILED-Egg graphic reported in Figure 11, which shows the predicted absorption of the described molecules by the BBB or by the gastrointestinal tract, or by neither of them. In contrast, we also report the oral bioavailability of our compounds based on the Lipinski rules of five. Unfortunately, for all our compounds, the efficacy and the bioavailability data do not correlate well and those molecules that can be orally administered also show blood–brain barrier (BBB) permeation, which could give rise to cytotoxic effects. Only compound **Fg-32** matches both requirements.

The graphical “BOILED-Egg” tool [39] easily allows for a comparison between the absorption pharmacodynamic of all families of compounds. This graph is based on the lipophilicity (WLogP) and apparent polarity (TPSA) of the different compounds. The yellow portion, which represents the yolk, contains those molecules whose physicochemical properties make them likely to permeate through the BBB. The white part contains those compounds that show a high probability of passive gastrointestinal absorption, while the outer grey area contains molecules featuring low absorption and limited brain penetration.

As we can observe, only seven molecules from a total of 41 show unsuitable drug-like properties. Compounds **Fa-M1**, **Fa-M2, Fa-M3**, **Fg-10**, and **Ff-1** are predicted to be able to cross the BBB, which is highly undesirable. Compounds **Fg-17** and **Fg-13** are almost at the limit towards low permeability and could cause absorption problems in further development stages. Additionally, compounds **Fg-11** and **Fg-9** are close to the BBB passive permeation area and, as such, could be inadequate for administration. The rest of the compounds show good physicochemical properties to be passively absorbed in the gastrointestinal tract. Moreover, they are all represented with a blue dot, which means that they are actively effluxed by the P-glycoprotein (PgP) in both the BBB and the GI tract according to the predictions. The PgP pumps out substrates/drugs, often becoming the main reason for drug resistance in cancer cells. Hence, even though further studies should be carried out to experimentally prove the predictions, this potential drawback should be taken into consideration [40].

## 4. Conclusions

In this work, we presented a series of compounds in which the cationic heads that, so far, have been used in CKα1 inhibitors were replaced by thienopyridine- and pyrimidine-derived bioisosters.

The most interesting aspect of this change is that it led to an overall remarkable increase in the inhibitory activity of the enzyme, independently of the linker used. More specifically, seven compounds showed an IC_50_ value < 2 μM. This highlights the suitability of these heads compared to those already used, which were featuring a pyridinic or quinolinic structure.

In terms of antiproliferative activity, the families with higher lipophilicity or longer linkers show better anti-tumor activity, with the most active being compound **Ff-35**: ((1,1′-((butane-1,4-diylbis(4,1-phenylene))bis(methylene))bis(4-(azepan-1-yl)thieno[2,3-d]pyrimidin-1-ium)) bromide. This compound also shows good enzyme inhibition (IC_50_ = 0.46 μM) and the ability to inhibit choline uptake with remarkable potency, suggesting a dualistic mode of action for its antiproliferative activity.

We also showed that **Ff-35** arrests the cell cycle in G1 phase, displaying a significant increase in apoptotic cell numbers in all three cell lines investigated. Interestingly, this occurs in a concentration-dependent manner, in good agreement with the cytotoxicity data.

Finally, the study of the predicted parameters related to the ADME suitability of the synthesized compounds determined that for **Ff-35** no PAINS were detected; thus, excluding the possibility of side effects due to the toxicity of chemical fragments within the cellular medium. Hence, the replacement of the old cationic heads by thienopyrimidine derivatives could represent a fresh starting point in the design of new CKα1 inhibitors with enhanced activity.

## Figures and Tables

**Table 1:** Compounds synthetized and evaluated. The different bioisosteric cationic heads are shown in blue, black, and red.

Compound		Family	Linker (a–f)	Bioisosteric Cationic Head	7 or 4 Substituent
**Fa-M2**	Monocationic	A	Biphenyl	thieno[3,2-b]pyridin-1-ium	Pyrrolidinyl
**Fa-M1**				thieno[3,2-d]pyrimidin-1-ium	
**Fa-M3**				thieno[2,3-d]pyrimidin-1-ium	
**Fg-9**	Biscationic	B	Biphenyl	thieno[3,2-b]pyridin-1-ium	Pyrrolidinyl
**Fa-21**				thieno[3,2-d]pyrimidin-1-ium	
**Fg-14**				thieno[2,3-d]pyrimidin-1-ium	
**Fa-24**				thieno[3,2-d]pyrimidin-1-ium	Piperidinyl
**Fg-30**				thieno[2,3-d]pyrimidin-1-ium	
**Fg-10**				thieno[3,2-b]pyridin-1-ium	Azepanyl
**Fa-22**				thieno[3,2-d]pyrimidin-1-ium	
**Fg-18**				thieno[2,3-d]pyrimidin-1-ium	
**Fp-1**				thieno[3,2-d]pyrimidin-1-ium	*N*-methyl-aniline
**Fp-8**				thieno[2,3-d]pyrimidin-1-ium	*p*-Chloro-*N*-methylaniline
**Fg-12**		C	Bipyridinyl	thieno[3,2-b]pyridin-1-ium	Pyrrolidinyl
**Fg-17**				thieno[3,2-d]pyrimidin-1-ium	
**Fg-13**				thieno[2,3-d]pyrimidin-1-ium	
**Fa-27**				thieno[3,2-d]pyrimidin-1-ium	Piperidinyl
**Fg-32**				thieno[2,3-d]pyrimidin-1-ium	
**Fa-26**				thieno[3,2-d]pyrimidin-1-ium	Azepanyl
**Fg-20**				thieno[2,3-d]pyrimidin-1-ium	
**Fg-11**		D	Bibenzyl	thieno[3,2-b]pyridin-1-ium	Pyrrolidinyl
**Fg-16**				thieno[3,2-d]pyrimidin-1-ium	
**Fg-15**				thieno[2,3-d]pyrimidin-1-ium	
**Fa-25**				thieno[3,2-d]pyrimidin-1-ium	Piperidinyl
**Fg-31**				thieno[2,3-d]pyrimidin-1-ium	
**Fa-23**				thieno[3,2-d]pyrimidin-1-ium	Azepanyl
**Fg-19**				thieno[2,3-d]pyrimidin-1-ium	
**Ff-1**		E	Biphenethyl	thieno[3,2-b]pyridin-1-ium	Pyrrolidinyl
**Ff-7**				thieno[3,2-d]pyrimidin-1-ium	
**Ff-3**				thieno[2,3-d]pyrimidin-1-ium	
**Fa-33**				thieno[3,2-d]pyrimidin-1-ium	Piperidinyl
**Ff-6**				thieno[2,3-d]pyrimidin-1-ium	
**Fa-29**				thieno[3,2-d]pyrimidin-1-ium	Azepanyl
**Ff-35**				thieno[2,3-d]pyrimidin-1-ium	
**Ff-2**		F	Diphenoxiethane	thieno[3,2-b]pyridin-1-ium	Pyrrolidinyl
**Ff-8**				thieno[3,2-d]pyrimidin-1-ium	
**Ff-4**				thieno[2,3-d]pyrimidin-1-ium	
**Fa-28**				thieno[3,2-d]pyrimidin-1-ium	Piperidinyl
**Ff-5**				thieno[2,3-d]pyrimidin-1-ium	
**Ff-34**				thieno[3,2-d]pyrimidin-1-ium	Azepanyl
**Ff-36**				thieno[2,3-d]pyrimidin-1-ium	

**Table 2:** Docking scores and calculated ΔG_binding_ of each compound.

Compound	% Inhibition at 10 µM	% Inhibition at 30 µM	4BR3		4CG8	
			g-score (kcal/mol)	ΔG_binding_ (kcal/mol)	g-score (kcal/mol)	ΔG_binding_ (kcal/mol)
**Fa-29**	100.00 ^a^	100.00 ^a^	−7.429	−90.93	−8.740	−116.31
**Fa-33**	98.90	97.80	−6.780	−70.10	−7.703	−86.74
**Ff-34**	94.31	99.02	−7.502	−107.36	−6.838	−108.16
**Fg-11**	93.07	94.25	−8.776	−114.95	−7.126	−97.43
**Ff-35**	90.55	97.80	−6.483	−95.67	−7.907	−96.44
**Ff-7**	89.95	95.88	−6.721	−87.32	−6.814	−92.68
**Fa-22**	89.59	97.15	−7.105	−112.67	−5.921	−88.89
**Fa-28 10**	87.69	92.19	−7.208	−103.95	−5.700	−93.52
**Fa-24**	84.64	98.40	−7.571	−109.75	−6.298	−106.62
**Ff-36**	84.47	96.07	−6.546	−108.85	−7.681	−87.98
**Fg-12**	83.33	98.61	−6.890	−89.43	−5.406	−82.50
**Ff-1**	82.15	90.83	−8.994	−103.45	−7.069	−81.64
**Fg-10**	80.76	96.98	−6.645	−97.26	−6.409	−116.55
**Fg-14**	80.28	94.14	−8.465	−119.33	−7.259	−98.01
**Fg-16**	80.28	85.87	−7.542	−122.68	−5.556	−83.41
**Ff-8**	79.23	92.88	−8.873	−113.72	−6.027	−83.02
**Ff-2**	77.41	82.77	−9.403	−117.89	−7.085	−104.19
**Fg-9**	75.81	94.95	−7.128	−103.77	−4.454	−109.32
**Fg-30**	75.46	85.71	−7.379	−118.12	−5.856	−79.10
**Fa-21**	74.57	86.58	−7.516	−119.75	−5.619	−68.03
**Ff-3**	74.17	80.09	−7.833	−99.93	−6.937	−93.24
**Fa-26**	73.33	85.09	−8.061	−114.66	−5.976	−91.59
**Fg-20**	71.88	80.83	−7.632	−111.57	−5.544	−73.41
**Fg-15**	71.11	81.29	−10.412	−131.90	−7.619	−102.47
**Fa-27**	70.28	81.49	−6.060	−72.32	−5.137	−75.34
**Fg-32**	65.38	67.57	−6.880	−78.31	−5.460	−69.69
**Ff-5**	64.21	85.71	−7.810	−114.51	−5.318	−55.38
**Ff-4**	55.23	77.28	−8.907	−124.18	−6.883	−97.60
**Fg-17**	54.90	60.95	−7.405	−113.16	−6.103	−77.09
**Fg-31**	51.54	74.39	−8.194	−118.41	−7.201	−95.92
**Ff-6**	46.43	73.19	−6.992	−76.19	−7.657	−109.86
**Fa-M1**	34.59	31.68	-6.978	−82.49	−5.331	−51.72
**Fa-M3**	34.42	37.94	−5.922	−82.47	−5.357	−68.05
**Fa-M2**	30.24	35.06	−6.308	−75.23	−5.755	−62.55
**Fg-13**	28.25	50.41	−6.513	−78.44	−5.294	−74.69
**Fa-23**	- ^b^	- ^b^	−7.346	−127.89	−7.105	−102.87
**Fa-25**	- ^b^	- ^b^	−8.089	−114.86	−7.912	−98.88
**Fg-18**	- ^b^	- ^b^	−7.376	−95.92	−8.146	−90.75
**Fg-19**	- ^b^	- ^b^	−8.567	−93.84	−5.755	−82.99

^a^ The calculated ΔG_binding_ was obtained by refining the best-scoring pose for each ligand. The percentage inhibition at 10 and 30 µM of **Fa-29** was considered to be 100% since at 5 µM it already showed a percentage inhibition of 85%. ^b^ The compound was not soluble.

**Table 3:** Selected examples of the reduction in the inhibitory potency in the thieno[2,3-d]pyrimidinium heads if compared to the thieno[3,2-d]pyrimidinium ones.

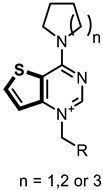		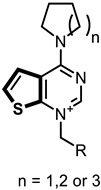	
Compound	% Inhibition at 10 µM	Compound	% Inhibition at 10 µM
**Fa-29**	100.00	**Ff-35**	90.55
**Fa-33**	98.9	**Ff-6**	46.43
**Fa-28**	87.69	**Ff-5**	64.21
**Ff-34**	94.31	**Ff-36**	84.47

**Table 4:** In vitro inhibitory effects of final compounds.

						GI_50_^c^ (µM)						
Comp.	Family	IC_50_ CKα ^a^	cLog P ^b^	HL-60	Jurkat	RS 4;11	SEM	A549	MDA-MB-231	HeLa	HT29	% Inhibition Choline Uptake ^d^
**Fa-M2**	A	≥50	3.47	0.13 ± 0.01	2.40 ± 0.22	0.56 ± 0.03	0.96 ± 0.01	3.5 ± 0.3	0.81 ± 0.01	0.38 ± 0.005	0.40 ± 0.02	
**Fa-M1**		≥50	3.00	0.15 ± 0.01	4.24 ± 0.32	0.68 ± 0.05	1.47± 0.2	0.39 ± 0.01	0.62 ± 0.03	0.41 ± 0.01	0.58 ± 0.02	−1 μM: 49.93 ± 1.72 −10 μM: 85.12 ± 1.35
**Fa-M3**		≥ 50	3.14	nd	Nd	nd	nd	nd	nd	nd	nd	
**Fg-9**	B	≥ 5	3.54	9.12 ± 1.1	>10	>10	>10	0.25 ± 0.02	2.02 ± 0.2	>10	>10	
**Fa-21**		≥5	2.75	3.40 ± 0.7	>10	>10	>10	0.22 ± 0.01	1.71 ± 0.4	>10	>10	
**Fg-14**		4.87 ± 0.57	2.81	1.35 ± 0.1	>10	>10	>10	0.28 ± 0.02	0.94 ± 0.09	2.53 ± 0.8	4.16 ± 1.0	
**Fa-24**		2.33 ± 0.21	2.93	7.28 ± 1.1	>10	>10	>10	1.17 ± 0.2	0.70 ± 0.07	7.08 ± 1.3	>10	
**Fg-30**		≥ 5	3.07	2,78 ± 0.5	>10	>10	>10	0.60 ± 0.02	0.80 ± 0.02	2.89 ± 0.3	8.32 ± 0.9	
**Fg-10**		≥ 10 μM	4.33	0.44 ± 0.05	6.33 ± 0.6	2.77 ± 0.9	3.51 ± 0.8	0.31 ± 0.01	1.46 ± 0.2	1.59 ± 0.1	1.76 ± 0.2	
**Fa-22**		2.87 ± 0.19	3.49	3.29 ± 0.5	>10	4.29 ± 0.7	>10	0.97 ± 0.02	1.42 ± 0.01	6.55 ± 0.4	6.51 ± 0.6	−0.1 μM: -1.15 ± 1.2 −0.5 μM: 60.6 ± 0.02 −2 μM: 90.08 ± 0.24
**Fg-18**		---	3.72	0.79 ± 0.02	7.04 ± 0.8	2.65 ± 0.04	>10	0.21 ± 0.01	0.61 ± 0.03	1.81 ± 0.02	2.32 ± 0.02	
**Fp-1**		1.06 ± 0.22	3.78	0.51 ± 0.05	>10	2.12 ± 0.2	4.55 ± 0.5	0.21 ± 0.02	0.90 ± 0.02	1.66 ± 0.01	1.43 ± 0.03	
**Fp-8**		---	5.12	0.90 ± 0.08	>10	3.38 ± 0.05	>10	0.16 ± 0.01	0.83 ± 0.04	1.77 ± 0.1	3.93 ± 0.5	
**Fg-12**	C	---	2.23	>10	>10	>10	>10	0.19 ± 0.01	0.80 ± 0.02	>10	>10	
**Fg-17**		≥25	1.32	>10	>10	>10	>10	0.14 ± 0.01	>10	>10	>10	
**Fg-13**		≥ 25	1.27	8.71 ± 1.2	>10	>10	>10	0.14 ± 0.01	>10	>10	>10	
**Fa-27**		≥5	1.54	>10	>10	>10	>10	1.06 ± 0.02	>10	>10	>10	
**Fg-32**		≥ 5	1.66	>10	>10	>10	>10	0.29 ± 0.02	>10	>10	>10	
**Fa-26**		≥5	1.97	>10	>10	>10	>10	0.23	>10	>10	>10	
**Fg-20**		≥ 5	2.18	1.22 ±	>10	>10	>10	>10	>10	>10	>10	
**Fg-11**	D	2.33 ± 0.20	3.76	0.47 ± 0.03	8.53 ± 1.1	5.72 ± 0.5	>10	0.39 ± 0.04	0.68 ± 0.07	2.19 ± 0.3	3.65 ± 0.5	
**Fg-16**		1.81 ± 0.17	2.93	0.21 ± 0.03	>10	7.27 ± 0.8	>10	0.25 ± 0.02	0.38 ± 0.01	0.67 ± 0.02	2.64 ± 0.06	
**Fg-15**		≥ 5	2.99	0.13 ± 0.01	>10	3.71 ± 0.04	>10	0.22 ± 0.03	0.26 ± 0.02	1.00 ± 0.1	2.01 ± 0.03	
**Fa-25**		---	3.35	0.14 ± 0.01	5.48 ± 0.5	1.84 ± 0.1	2.53 ± 0.03	0.28 ± 0.02	0.33 ± 0.02	0.62 ± 0.02	1.47 ± 0.06	
**Fg-31**		≥ 10	3.43	0.76 ± 0.05	>10	7.20 ± 1.0	>10	1.17 ± 0.01	1.09 ± 0.1	1.40 ± 0.02	4.50 ± 0.4	
**Fa-23**		---	3.95	0.51 ± 0.05	6.15 ± 0.6	2.08 ± 0.03	2.17 ± 0.02	0.60 ± 0.06	1.13 ± 0.2	1.17 ± 0.1	1.56 ± 0.2	
**Fg-19**		---	4.15	0.15 ± 0.01	4.72 ± 0.6	1.51 ± 0.2	2.59 ± 0.03	0.31 ± 0.04	0.50 ± 0.02	0.78 ± 0.02	1.42 ± 0.1	
**Ff-1**	E	1.54 ± 0.17	4.42	0.79 ± 0.05	4.13 ± 0.4	7.90 ± 1.3	3.07 ± 0.3	0.97 ± 0.02	1.23 ± 0.01	1.90 ± 0.01	3.32 ± 0.03	
**Ff-7**		8.57 ± 2.80	3.59	0.40± 0.05	8.61 ± 1.3	2.33 ± 0.3	2.93 ± 0.2	0.21 ± 0.03	0.47 ± 0.04	0.87 ± 0.05	1.48 ± 0.04	
**Ff-3**		≥ 5	3.80	0.21 ± 0.03	7.54 ± 1.5	2.05 ± 0.1	3.70 ± 0.4	0.21 ± 0.02	0.36 ± 0.03	0.63 ± 0.02	1.33 ± 0.01	
**Fa-33**		1.67 ± 0.08	4.09	0.14 ± 0.01	1.46 ± 0.1	0.76 ± 0.1	1.37 ± 0.07	0.16 ± 0.01	0.41 ± 0.02	0.28 ± 0.02	1.07 ± 0.1	
**Ff-6**		≥ 10	4.21	0.19 ± 0.01	6.7 ± 0.8	1.50 ± 0.2	2.52 ± 0.2	0.19 ± 0.03	0.50 ± 0.02	0.42 ± 0.05	0.93 ± 0.04	
**Fa-29**		1.08 ± 0.07	4.81	0.55± 0.02	5.23 ± 0.8	1.60 ± 0.1	1.77 ± 0.2	0.14 ± 0.02	0.50 ± 0.02	0.84 ± 0.04	1.09 ± 0.2	
**Ff-35**		0.46 ± 0.08	4.91	0.15 ± 0.01	1.54 ± 0.02	0.95 ± 0.05	0.76 ± 0.03	0.14 ± 0.02	0.097 ± 0.001	0.30 ± 0.01	0.78 ± 0.03	−0.1 μM: 35.64 ± 0.7 −0.5 μM: 84.99 ± 1.3 −2 μM: 96.62 ± 0.316
**Ff-2**	F	nd	3.14	2.14 ± 0.1	>10	>10	>10	1.06 ± 0.1	1.43 ± 0.2	3.26 ± 0.4	>10	
**Ff-8**		nd	2.28	0.55 ± 0.02	>10	3.47 ± 0.3	>10	0.29 ± 0.3	0.32 ± 0.05	0.91 ± 0.09	1.89 ± 0.2	
**Ff-4**		≥10	2.50	0.79 ± 0.05	>10	>10	>10	0.23 ± 0.03	0.72 ± 0.03	>10	>10	
**Fa-28**		3.25 ± 0.54	2.78	0.35 ± 0.01	>10	2.32 ± 0.03	2.41 ± 0.04	0.29 ± 0.02	0.19 ± 0.01	0.59 ± 0.02	>10	
**Ff-5**		≥5	2.94	0.20 ± 0.01	>10	2.45 ± 0.02	3.27 ± 0.03	0.34 ±0.06	1.10 ± 0.2	1.03 ± 0.1	1.73 ± 0.3	
**Ff-34**		0.70 ± 0.03	3.48	0.27 ± 0.01	6.1 ± 1.1	1.8 ± 0.1	2.5 ± 0.1	0.29 ± 0.03	0.91 ± 0.08	0.93 ± 0.01	1.63 ± 0.1	
**Ff-36**		0.35 ± 0.05	3.69	0.44 ± 0.02	6.2 ± 0.9	2.26 ± 0.3	2.54 ± 0.2	1.43 ± 0.1	2.26 ± 0.3	3.02 ± 0.4	>10	−0.5 μM: 71.78 ± 0.1 −2 μM: 95.32 ± 0.29
**MN-48b ** ^**d**^		0.78 ± 0.03	1.27	0.32 ± 0.03	0.35 ± 0.1	1.0 ± 0.3	nd	0.54 ± 0.2	0.31 ± 0.12	1.9 ± 0.1	1.9 ± 0.4	
**RSM-932A ** ^**d**^		1.92 ± 0.06	4.13	0.93 ± 0.1	0.41 ± 0.1	0.17 ± 0.04	nd	0.45 ± 0.09	0.17 ± 0.05	0.83 ± 0.1	0.4 ± 0.2	

^a^ IC_50_ = compound concentration required to inhibit CKα1 enzyme by 50%. ^b^ The values of clogP were calculated with the free website tool http://www.swissadme.ch/ (last access the 18 November 2021). ^c^ GI_50_ = compound concentration required to inhibit tumor cell proliferation by 50%. ^d^ Values of percentage of inhibition of choline uptake in HepG2. nd = not determined.

**Table 5:** In silico predicted physicochemical, drug-likeness, and medicinal chemistry adequacy features for all final compounds.

	Compound	cLog P	PAINS	Mw	Structural Alert	Lipinski Rules	Suitability
Monocationic Biphenyl	Fa-M2	3.47	NO	451.42	Quaternary N	Yes	No
	Fa-M1	3.00	NO	452.41	Quaternary N	Yes	No
	Fa-M3	3.14	NO	452.41	Quaternary N	Yes	No
Biscationic	Fg-9	3.54	NO	748.64	Quaternary N	No	Moderate
Biphenyl	Fa-21	2.75	NO	750.62	Quaternary N	No	Yes
	Fg-14	2.81	NO	750.62	Quaternary N	No	Yes
	Fa-24	2.93	NO	778.67	Quaternary N	No	Yes
	Fg-30	3.07	NO	778.67	Quaternary N	No	Yes
	Fg-10	4.33	NO	804.75	Quaternary N	No	No
	Fa-22	3.49	NO	806.72	Quaternary N	No	Yes
	Fg-18	3.72	NO	806.72	Quaternary N	No	Yes
	Fp-1	3.78	NO	822.68	Quaternary N	No	Yes
	Fp-8	5.12	NO	891.57	Quaternary N	No	Yes
Bipyridinyl	Fg-12	2.23	NO	750.62	Quaternary N	No	Yes
	Fg-17	1.32	NO	752.59	Quaternary N	Yes	No
	Fg-13	1.27	NO	752.59	Quaternary N	Yes	No
	Fa-27	1.54	NO	780.65	Quaternary N	No	Yes
	Fg-32	1.66	NO	780.65	Quaternary N	Yes	Yes
	Fa-26	1.97	NO	808.7	Quaternary N	No	Yes
	Fg-20	2.18	NO	808.7	Quaternary N	No	Yes
Bibenzyl	Fg-11	3.76	NO	776.69	Quaternary N	No	Moderate
	Fg-16	2.93	NO	778.67	Quaternary N	No	Yes
	Fg-15	2.99	NO	778.67	Quaternary N	No	Yes
	Fa-25	3.35	NO	806.72	Quaternary N	No	Yes
	Fg-31	3.43	NO	806.72	Quaternary N	No	Yes
	Fa-23	3.95	NO	834.78	Quaternary N	No	Yes
	Fg-19	4.15	NO	834.78		No	Yes
Biphenethyl	Ff-1	4.42	NO	804.75	Quaternary Quaternary NN	No	No
	Ff-7	3.59	NO	806.72	Quaternary N	No	Yes
	Ff-3	3.80	NO	806.72	Quaternary N	No	Yes
	Fa-33	4.09	NO	834.78	Quaternary N	No	Yes
	Ff-6	4.21	NO	834.78	Quaternary N	No	Yes
	Fa-29	4.81	NO	862.83	Quaternary N	No	Yes
	Ff-35	4.91	NO	862.83	Quaternary N	No	Yes
Diphenoxiethane	Ff-2	3.14	NO	808.69	Quaternary N	No	Yes
	Ff-8	2.28	NO	810.67	Quaternary N	No	Yes
	Ff-4	2.50	NO	810.67	Quaternary N	No	Yes
	Fa-28	2.78	NO	838.72	Quaternary N	No	Yes
	Ff-5	2.94	NO	838.72	Quaternary N	No	Yes
	Ff-34	3.48	NO	866.78	Quaternary N	No	Yes
	Ff-36	3.69	NO	866.78	Quaternary N	No	Yes

## Data Availability

Not applicable.
